# Developing a 3D bone model of osteosarcoma to investigate cancer mechanisms and evaluate treatments

**DOI:** 10.1096/fj.202402011R

**Published:** 2024-12-26

**Authors:** Hannah L. Smith, Stephen A. Beers, Janos M. Kanczler, Juliet C. Gray

**Affiliations:** ^1^ Antibody and Vaccine Group, Faculty of Medicine, Centre for Cancer Immunology, School of Cancer Sciences University of Southampton Southampton UK; ^2^ Bone and Joint Research Group, Human Development and Health, Faculty of Medicine, Institute of Developmental Sciences University of Southampton Southampton UK; ^3^ Institute for Life Sciences University of Southampton Southampton UK

**Keywords:** 3D model, bone, bone marrow stromal cells, macrophage, osteosarcoma

## Abstract

Osteosarcoma is the most common primary bone cancer, occurring frequently in children and young adults. Patients are treated with surgery and multi‐agent chemotherapy, and despite the introduction of mifamurtide in 2011, there has been little improvement in survival for decades. 3‐dimensional models offer the potential to understand the complexity of the osteosarcoma tumor microenvironment and aid in developing new treatment approaches. An osteosarcoma 3D bone core model was developed using human trabecular bone and the chorioallantoic membrane (CAM), to form a functioning vasculature. A tri‐culture of cells, stromal cells, macrophages, and the Saos‐2 osteosarcoma cell line, were implanted into this model to simulate components of the tumor microenvironment, and mifamurtide was tested in this context. Immunohistochemistry and micro‐CT were performed to assess phenotypic and structural effects of implantation. Successful integration and angiogenesis of the bone cores were observed after incubation on the CAM. The 3D bone model also showed similar characteristics to osteosarcoma patient samples including CD68 and CD105 expression. Incubating bone cores with mifamurtide induced a reduction of cellular markers and an increase in bone volume. This 3D bone core model has the potential to investigate osteosarcoma tumor microenvironment and provides a representative model for evaluation of novel therapies.

## Graphical abstract

Bone cores were removed from human femoral heads, impregnated with cells and incubated on the CAM for 11 days. IHC and μCT were used to analyze changes in bone formation and phenotype.

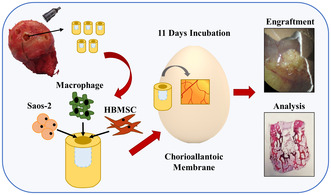

## 1 INTRODUCTION

Osteosarcoma, although a rare type of cancer, is the most frequent primary bone tumor in children and adolescents. It most commonly arises in the metaphyses of long bones, including the proximal tibia, humerus, and distal femur.^1^ In healthy bone, there is a regulated cycle of bone formation and resorption, but in the osteosarcoma microenvironment this process is disrupted by tumor cells to promote growth and metastasis. It is common for osteosarcoma patients to have both areas of random bone formation, as well as regions of excessive bone resorption as the tumor evolves to need more space.^2^ The drug mifamurtide is an immunomodulating liposome encapsulated muramyl tripeptide (L‐MTP‐PE) and has been approved in Europe as a clinical treatment of osteosarcoma since 2011.^3^ This drug is given concurrently with post‐operative chemotherapy with the aim of eradicating residual micro‐metastases.^4^ The mechanism of action of mifamurtide, although still unclear, is proposed to be through activation of an immune response via phagocytic cells.^5^ Unfortunately, even with the addition of mifamurtide, outcomes for osteosarcoma have been largely unchanged over several decades, necessitating the need for novel therapeutic drug strategies.

Research using 2‐dimensional (2D) in vitro assays have been essential in the field of oncology; producing novel insights into protein expression, cell biology, and the cellular morphology of different cancer types.^6^ However, they have many limitations including inducing artificial changes in morphology, as well as inhibiting cellular and extracellular interactions.^6^ Increasing awareness of the high level of heterogeneity and complexity of the tumor microenvironment has led to the development of robust and informative 3‐dimensional (3D) models better able to reproduce the cellular interactions found in the tumor microenvironment. These 3D models offer the potential to better understand micro‐environmental interactions particularly in the bone microenvironment where mechanical signals are very important in modulating tumor behavior.^7^ In vivo mouse models have and are being utilized to investigate osteosarcoma, with subcutaneous or intraosseous injection of osteosarcoma cell lines^8^, ^9^ being used effectively to screen drugs and assess migration.^10^, ^11^ However, while mouse models do offer the potential to gain insight into the development and characterization of osteosarcoma, their limited representation of the human disease due to species differences, alongside high cost and skilled procedures required to generate these models, suggests more robust human alternatives are needed.

One potential alternative model is through utilizing the chorioallantoic membrane (CAM) of a fertilized chicken egg. The CAM contains a dense vascular network that rapidly develops in the egg with its main role as a respiratory organ for the embryo, storing waste products and absorbing calcium from the shell.^12^ A distinctive property of the CAM is its natural immunodeficiency which means that host avian cells do not reject or majorly alter implanted tissue,^13^ allowing for in‐depth phenotypic analysis over prolonged periods. One of the most important benefits of CAM models over 2D methods is that it allows for the study of angiogenesis. Angiogenesis promotes the generation of blood vessels, which is important in tumor development.^14^ Three human osteosarcoma cell lines have previously been shown to form solid vascular tumors when inserted on the CAM, including the osteosarcoma‐derived cell line Saos‐2.^12^ This cell line has been well characterized both in vitro^15^ and *in ovo*.^12^ Unfortunately, current published studies of osteosarcoma in this context have been limited to cells impregnated in sponges,^16^ to add a scaffold for tumor development, or those implanted directly onto the CAM.^12^, ^17^ Combining osteosarcoma cell lines with structural human bone tissue on the angiogenic CAM could generate a better model to replicate the osteosarcoma environment and provide enhanced insights into cellular processes and interactions.

## 2 MATERIALS AND METHODS

### 2.1 Ethical approval

For all human tissue used, informed patient consent was obtained in alignment with the Declaration of Helsinki. Ethical approval was obtained for using human leukocyte cones (REC number 16/ES/0048), bone marrow and femoral head samples (REC number 18/NM/0231), and osteosarcoma patient samples (REC number 10/H0504/32). All CAM procedures were carried out under a ASPA approved Home Office Project license (P3E01C456).

### 2.2 Cell isolation and culture

Human bone marrow stromal cells (HBMSCs) were isolated and cultured as previously published.^15^ These cells were passaged for a maximum of three times. The osteosarcoma cell line Saos‐2 (ATCC, Virginia, USA, RRID:CVCL_0548) was cultured in complete alpha‐MEM media (c.αMEM) at 37°C in a humidified 5% CO_2_/balanced air incubator, and routinely tested for mycoplasma contamination. Monocyte‐derived macrophages (MDMs) were differentiated from PBMC leukocyte cones as previously described^18^; the cells were differentiated in alpha‐MEM (Lonza, Basel, Switzerland, BE02‐002F) + 10% FCS (Sigma, Burlington, USA, F4135) + 1% PS (Lonza, Basel, Switzerland, DE17‐602E) with 100 ng/mL human macrophage colony‐stimulating factor (M‐CSF, produced in house) for 7 days before inclusion in the model.

### 2.3 Generating the bone core model

Human femoral heads represent a suitable and available source of skeletal material and were obtained from osteoarthritic or osteoporotic patients undergoing elective hip replacement surgery. Bone cores were generated from femoral heads using a method adapted from a previously established protocol.^19^ Briefly, a hole saw dental drill bit was used to create 8 mm cores from femoral head samples with a partial defect inserted through the center of the bone core. For each core, 20 μL of cell suspension was injected into the defect area. This 20 μL cell suspension consisted of 6 × 10^5^ Saos‐2 cells, 6 × 10^5^ MDMs and 1.8 × 10^5^ HBMSCs that were either combined or individually seeded into the bone core model. Cell numbers were selected to ensure engraftment of a significant population of each cell type. The number of HBMSCs used in this model was lower than that compared to Saos‐2 and MDM due to the ability of skeletal progenitor cells to rapidly proliferate under injury‐induced conditions.^20^, ^21^ The cell suspension was resuspended in 11 μg/mL (w/v) Alginate (Sigma, Burlington, USA, A1112) + 20 μg/mL (w/v) Gelatine (Sigma, Burlington, USA, G1890) prior to insertion into the bone core. After a 2‐h incubation the bone cores were either inserted onto the CAM or incubated in complete alpha‐MEM media at 37°C in a humidified 5% CO_2_/balanced air incubator (standard cell/organ culture).

### 2.4 Chorioallantoic membrane

Fertilized wild type chicken eggs (Henry Stewart & Co, Norfolk, UK) were placed in an incubator (Hatchmaster, Brinsea, UK) for 7–8 days at 37°C in a 60% humidified atmosphere, with rotation every hour. At day 7 (age since fertilization), a 1 cm^2^ window was cut into each eggshell using a scalpel, and one implant placed on each membrane. Bone cores were allocated randomly to fertilized eggs and given arbitrary numbers for identification. Parafilm that had been previously sterilized in 70% (v/v) ethanol was used to seal the windows. The eggs were returned to the incubator for a further 11 days without rotation. On day 18, the windows were widened, and the bone cores cut away from the CAM. All chicken embryo studies and euthanasia were performed in accordance with UK Home Office approved methods. Five bone cores/eggs were allocated to each condition to allow for potential unfertilized eggs or lack of embryo development, if this occurred these bone cores were excluded from analysis. Less than 10% of total eggs used were excluded from analysis.

### 2.5 Histology

After incubation, the cores were fixed with 4% (w/v) paraformaldehyde (PFA, prepared in house) for 24–72 h, before being decalcified in 6% (v/v) trichloroacetic acid (TCA, Sigma, Burlington, USA, T6399) in deionized H_2_O for up to 7 days. The bone cores were then embedded in OCT (CellPath, Powys, UK, KMA‐0100‐00A) and placed in a vacuum (Welch 2511 dry vacuum) for 20 min before freezing on dry ice. 10 μm sections were cut using a cryostat (Leica CM1850) and transferred onto TOMO® adhesive microscope slides (CellPath, Powys, UK, MBE‐0302‐02A).

### 2.6 Immunohistochemistry

Slides were incubated in acetone for 10 min at 4°C, rehydrated in 1x PBS for 10 min before being stained for human CD68 (Agilent, California, USA, M087601‐2), CD105 (Abcam, Cambridge, UK, ab231774) or RANK (Novus Biologicals, Missouri, USA, NBP2‐24702) using the ImmPRESS Horse anti‐Rabbit (MP 7801‐15) and Horse anti‐mouse (MP 5402‐15) polymer staining kits (Vector Laboratories, California, USA). The kits were used following the manufacturers protocol, before being counterstained with light green SF solution. For antigen retrieval, the slides were incubated at 90°C for 20 min in either EDTA or citrate solution. EDTA: 0.37 g EDTA in 1 L H_2_O + 0.5 mL Tween‐20, pH8. Citrate: 3 g Sodium Citrate in 1 L H_2_O, pH6.

### 2.7 Hematoxylin and Eosin

Sections were rehydrated then incubated with Hematoxylin (Weigert's, solution A and B in equal measures, ClinTec, Rotherham, UK, 640495 and 640505) for 10 min, dipped in 1% (v/v) HCL in 70% (v/v) Ethanol five times, then stained with 1% (w/v) Eosin Y (Sigma, Burlington, USA, E6003) for 10 min. Slides were then washed and dehydrated before being mounted in DPX (Fisher Scientific, Pittsburgh, USA, D/5319/05). Images were taken using Zeiss Axiovert 200 microscope with Axiovision Software (Zeiss, Oberkochen, Germany).

### 2.8 Multiplex staining

Formalin‐fixed, paraffin‐embedded osteosarcoma patient samples were stained (using the antibodies above) by the Research Histology Department, University Southampton Hospital (an accredited pathology laboratory), using a Dako (AS4) multiplex staining machine and scanned at high resolution using an Axioscan (Zeiss, Oberkochen, Germany). The level of staining was quantified using Image J.

### 2.9 Image analysis

For each stained section from a bone core an average of five representative fields of view were acquired using a Zeiss Axiovert 200 microscope with Axiovision Software (Zeiss, Oberkochen, Germany, RRID:SCR_002677). The level of staining was quantified using Image J (RRID:SCR_003070); images were deconvoluted, separating the positive marker staining from the background stain. A threshold was set and used for all images in the same experiment. The resulting binary image was assessed for particle analysis, where the pixel area of positive stain and percentage area of positive stain was recorded.

### 2.10 Micro‐CT scanning

Micro‐computed tomography (μCT) images were taken of all cores before and after incubation using Bruker micro‐CT (Skyscan 1176, Massachusetts, USA) scanner. They were scanned in low density 1.5 mL Eppendorf tubes (Greiner Bio‐one, Stonehouse, UK, 616201) using the following settings: average voxel size 18 μm, X‐ray source 45 kV, 556 μA, Al 0.2 mm filter, rotation step 0.70° and exposure time 496 ms. Results were analyzed using NRecon; misalignment compensation (−2), ring artifact reduction (9) and beam‐hardening correction (40%), DataViewer; aligned under 3D registration, and CTAn; pixel of grayscale value between 70 to 255. A region of interest was also centered over the defect area with approximately 1 mm of edging into the core.

### 2.11 Statistics

Experimental data were analyzed using GraphPad Prism version 9.2 software. Results were expressed as mean ± SD. Significance was assessed using one‐way ANOVA (>2 groups) with Tukey's post hoc test. Statistical test used is stated on each figure. Values of *p* ≤ .05 were considered significant. Significance presented as *<.05, **<.01, ***<.001, ****<.0001.

## 3 RESULTS

### 3.1 Utilizing the CAM to develop a 3D bone model

**FIGURE 1:**
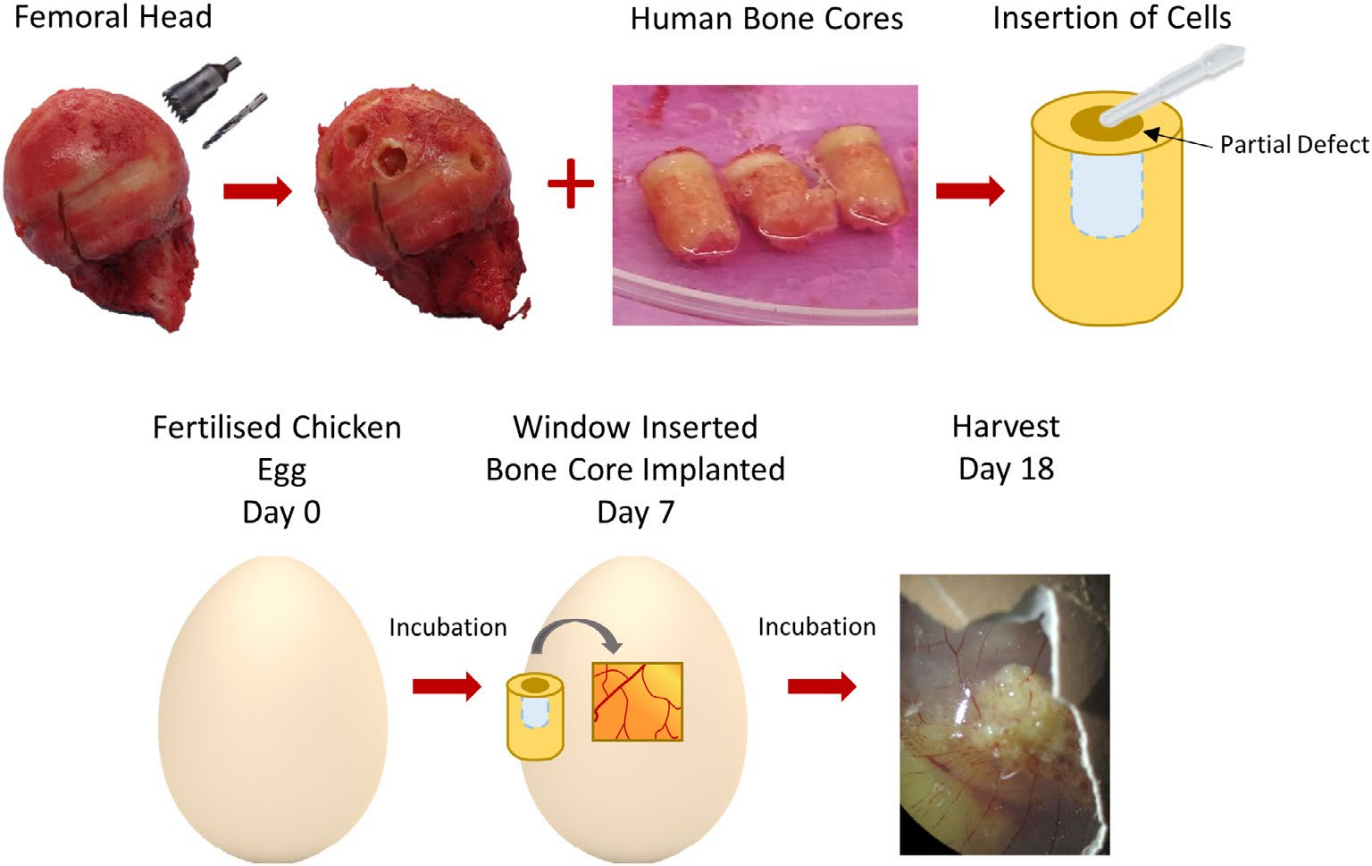
Development of the 3D bone core model. The bone cores were isolated from the femoral head of patients undergoing elective hip replacement surgery, and a partial defect was drilled into the center, which allowed for the insertion of cells. The bone cores were then implanted on the CAM of a chicken egg 7 days after fertilization and harvested on day 18.

To develop innovative treatments for osteosarcoma we need to gain a more comprehensive understanding of cellular dynamics in the tumor microenvironment. Here, we aimed to develop a 3D bone model which could be used to simulate these interactions and to test novel anti‐osteosarcoma agents. To do this, a previously established bone cylinder model^22^ which exhibited blood vessel infiltration, deposition of extracellular matrix and increased bone volume was adapted (Figure 1). The bone cores were incubated on the CAM to provide a functioning vasculature in order to replicate some of the complex cellular interactions found in the human tumor microenvironment, and to maintain viable tissue for long‐term culture.

**FIGURE 2:**
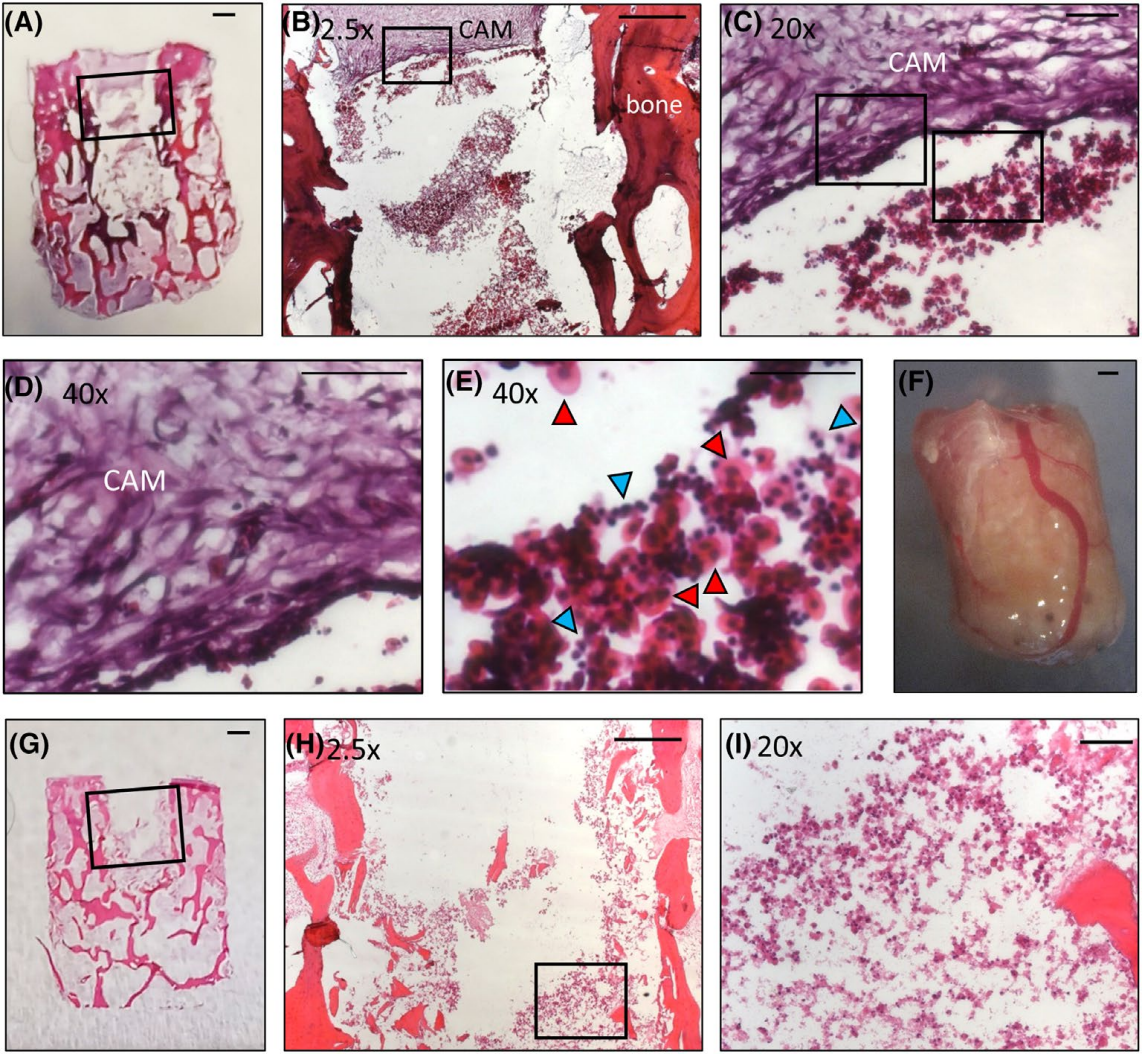
Vascularization of the 3D bone core model. Bone cores were inserted with a combination of Saos‐2, MDM and HBMSCs (SMH) and cultured on the CAM for 11 days. (A) Representative H+E staining of a bone core. Distinction between the CAM and the bone around the defect region in (B). A higher magnification of the defect region in (C) can identify the edge of the CAM (D). In (E) nucleated erythrocytes (red arrow) and thrombocytes (blue arrow) can be identified infiltrating into the defect region. (F) Blood vessel integration can still be seen surrounding the bone core after removal from the CAM. (G) Representative H+E staining of a bone core inserted with SMH cells and incubated in standard culture conditions for 11 days, with higher magnifications of the defect region shown in (H, I).

The osteosarcoma cell line Saos‐2 were included to represent osteosarcoma tumor cells in this bone core model. These cells were chosen as they have previously been demonstrated to form tumors *in ovo* without the invasive growth patterns and increased metastasis induced by other osteosarcoma cell lines (e.g. MNNH‐HOS).^23^ It was also important for macrophages to be incorporated as they are a prominent cell population identified in the osteosarcoma tumor microenvironment, accounting for approximately 50% of immune infiltration,^24^ although their clinical impact remains controversial.^25^ MDMs were isolated from PBMC cones and differentiated for 7 days before being included in the model. In previous studies,^15^ we have shown that bone marrow taken from different areas of the femur has differing characteristics. To replicate the osteosarcoma microenvironment more accurately, HBMSCs from the femoral diaphysis/metaphysis were included, as these have previously been shown to be enriched with hematopoietic cells.^15^ The bone cores were successfully engrafted into the CAM as shown in Figure 2. Here, a section of the CAM spans the top of the defect region (Figure 2A), and with higher magnification the edge of the CAM can be visualized (Figure 2B–D). Underneath the CAM, within the defect region, there is evidence of vascularization (Figure 2E) with both nucleated erythrocytes (red arrows) and thrombocytes (blue arrows) present in the bone cores. These findings support that the blood vessels clearly seen surrounding the bone cores (Figure 2F) also penetrate into the bone tissue. In contrast, there was no evidence of nucleated erythrocytes or thrombocytes in bone cores incubated in standard culture conditions (Figure 2G–I).

**FIGURE 3:**
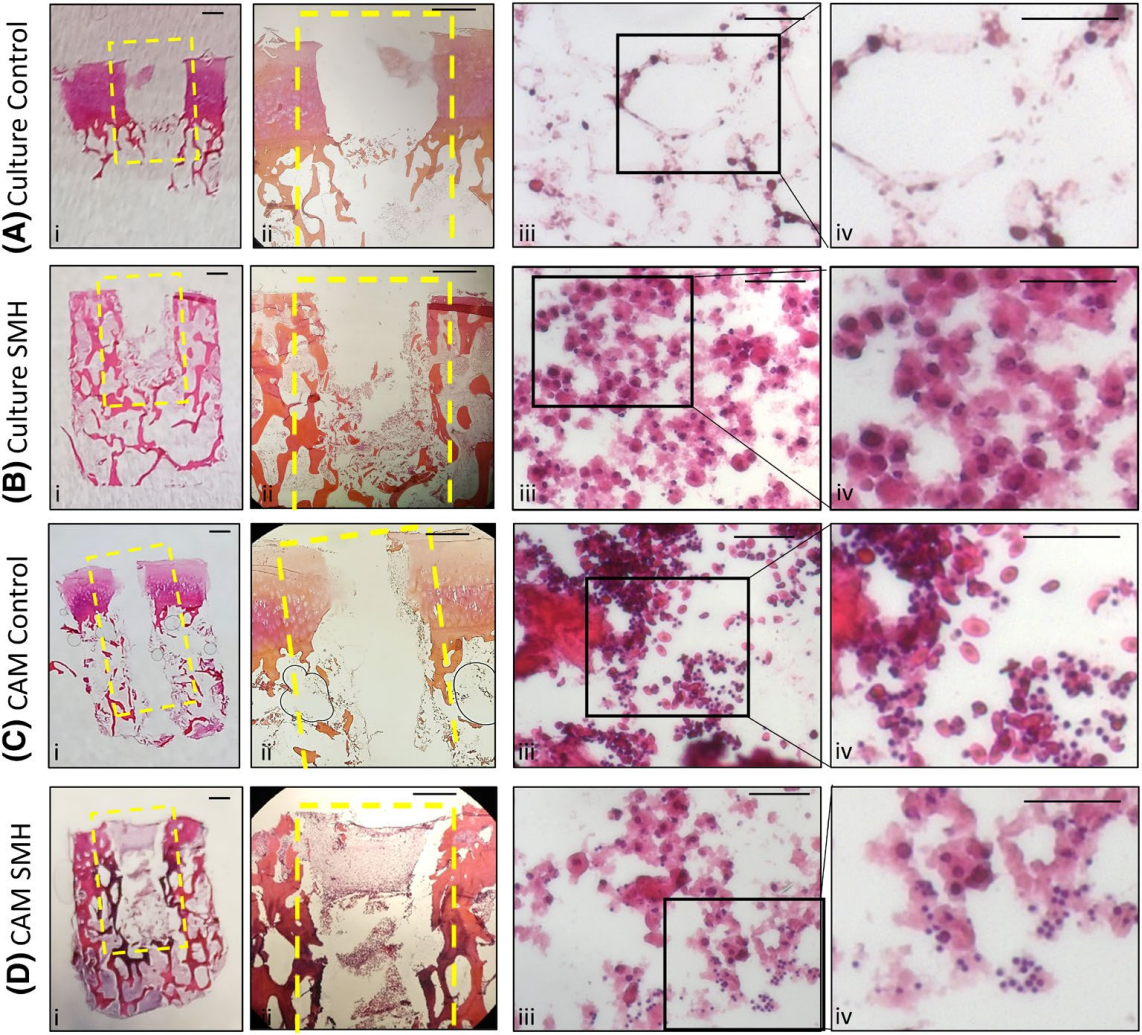
Assessment of the 3D Bone Core Model. Bone Cores were isolated from human femoral heads, and a defect was drilled into the center, highlighted in yellow. The bone cores were either incubated in standard culture conditions alone (A—control) or after insertion of Saos‐2, MDMs and HBMSCs (B—SMH), inserted onto the CAM alone (C—control) or after insertion of SMH (D). For representative images of HE stained bone cores (A–D i and ii) the scale bar = 1 mm. For representative H+E staining from the defect region of the bone cores identifying inserted SMH cells, infiltrating chick erythrocytes and thrombocytes (A–D iii and iv) the scale bar = 100 μm.

For all bone cores generated, the inserted cells remained within and around the defect area (Figure 3i,ii, highlighted in yellow). A clear difference in morphology can be observed between the cells in the defect region and the surrounding bone matrix, recreating the ‘tumor niche’. Resident bone marrow cells, visible in Figure 3Aiii,iv have a different morphology and density compared to cells inserted into the defect region in Figure 3Biii,iv, which were injected with a combination of Saos‐2 cells, MDMs and HBMSCs (abbreviated to SMH). When incubated on the CAM, cells in the defect region of the control cores (Figure 3C) consisted of nucleated erythrocytes and thrombocytes as described earlier. These cells can be observed in close proximity with the inserted SMH populations in Figure 3D.

### 3.2 Characterization of the 3D osteosarcoma bone model

**FIGURE 4:**
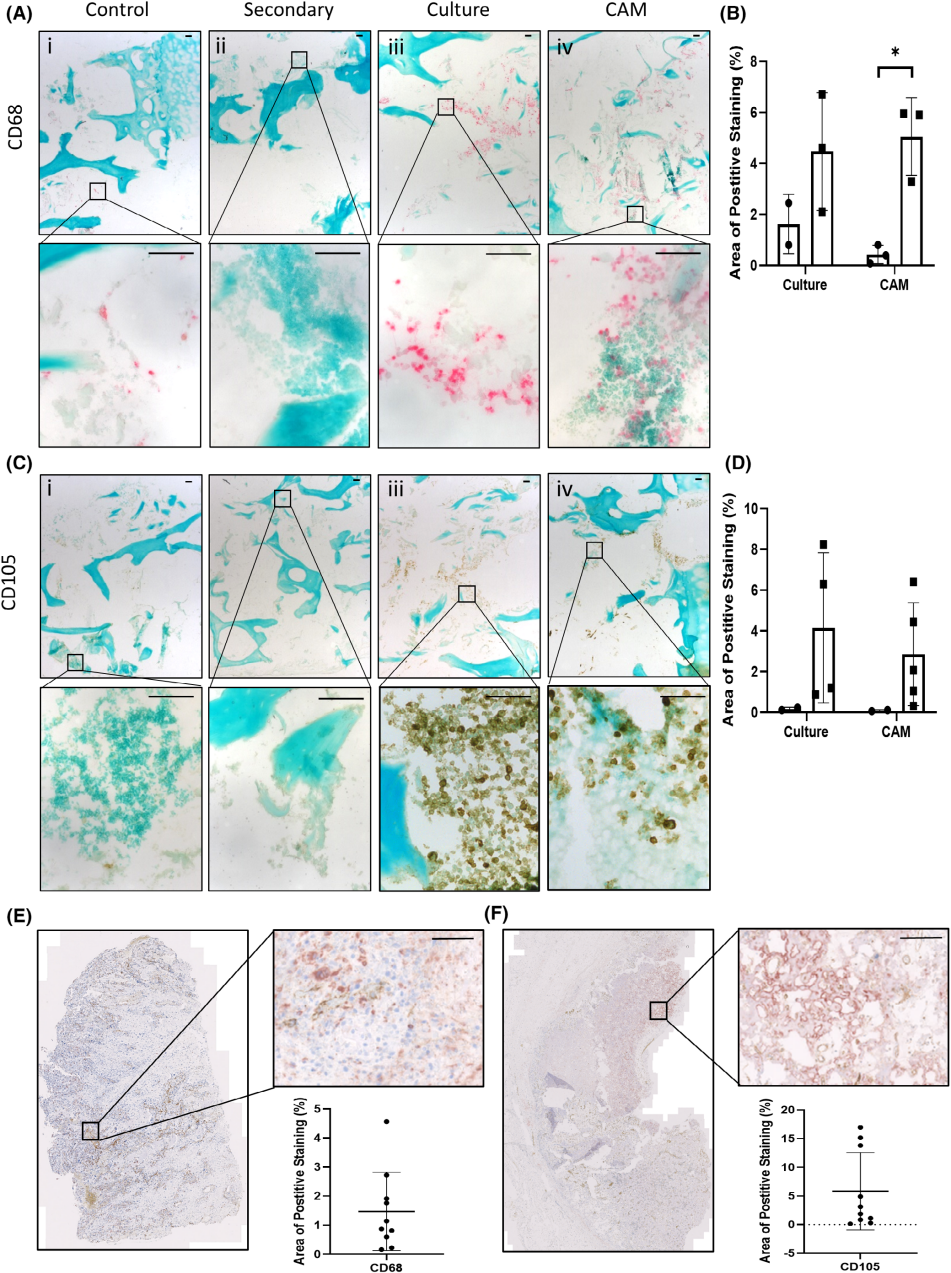
CD68 and CD105 expression of bone cores after incubation in standard culture or implantation on the CAM. A combination of Saos‐2, MDMs and HBMSCs (abbreviated to SMH) were inserted into the bone cores, which were then incubated for 11 days before being fixed, decalcified in 6% TCA, and embedded in OCT. 10 μm sections were stained (A) and quantified (B) for percentage area staining for CD68 using Image J, (i) control = cores with no cells, (ii) secondary = secondary only staining, (iii) incubated in standard culture conditions, (iv) incubated on the CAM. Representative images of CD105 staining (C) were also quantified (D) for percentage area staining. *N* = 2–5 biological replicates. Results presented as mean ± SD, statistics analyzed using a one‐way ANOVA, significance presented as *<.05. Representative images of osteosarcoma patient tissue stained and quantified for (E) CD68 and (F) CD105. *N* = 10. For all images scale bar = 100 μm.

After identifying three cell types to be included in the 3D bone model, Saos‐2 cells, MDMs and HBMSCs, the cells were combined, inserted into the bone cores and either incubated on the CAM or in complete alpha‐MEM media in standard culture for 11 days as indicated in materials and methods. After the incubation period, the bone cores were fixed, decalcified, and embedded in OCT. After sectioning, immunohistochemistry (IHC) staining was performed on the bone cores for the expression of the macrophage marker CD68.^26^ Figure 4A are representative images of CD68 staining of (i) unseeded control, (ii) secondary only staining, (iii) standard culture conditions, and (iv) CAM incubated bone cores, with the combined area of positive staining quantified in Figure 4B. The bone cores were also stained for CD105, a marker of endothelial and mesenchymal stem cells.^27^, ^28^ This marker was used to identify both inserted stromal cells as well as the presence of blood vessels. Figure 4C are representative images of CD105 staining, with the combined area of positive staining quantified in Figure 4D. For both CD68 and CD105 there were similar levels of staining found between the bone cores incubated in culture and on the CAM. Although there was positive staining found throughout the bone cores, the strongest staining was located within the defect area. This supported the proposition that there was limited migration of cells outside the defect region. These observations identified successful cellular integration of the CAM incubated bone cores, with no detriment to the viability of the inserted cells compared to standard in vitro conditions. Ten osteosarcoma patient samples were also stained in a multiplex machine for the same markers, and showed a range in expression of both CD68 (Figure 4E) and CD105 (Figure 4F) staining. The percentage positive staining identified within the bone cores was found to be within the same range, observed in the ten osteosarcoma samples. These data support the ability of the bone core model to mimic clinically relevant aspects of the cellular osteosarcoma microenvironment.

### 3.3 Bone remodeling of the 3D osteosarcoma bone model

**FIGURE 5:**
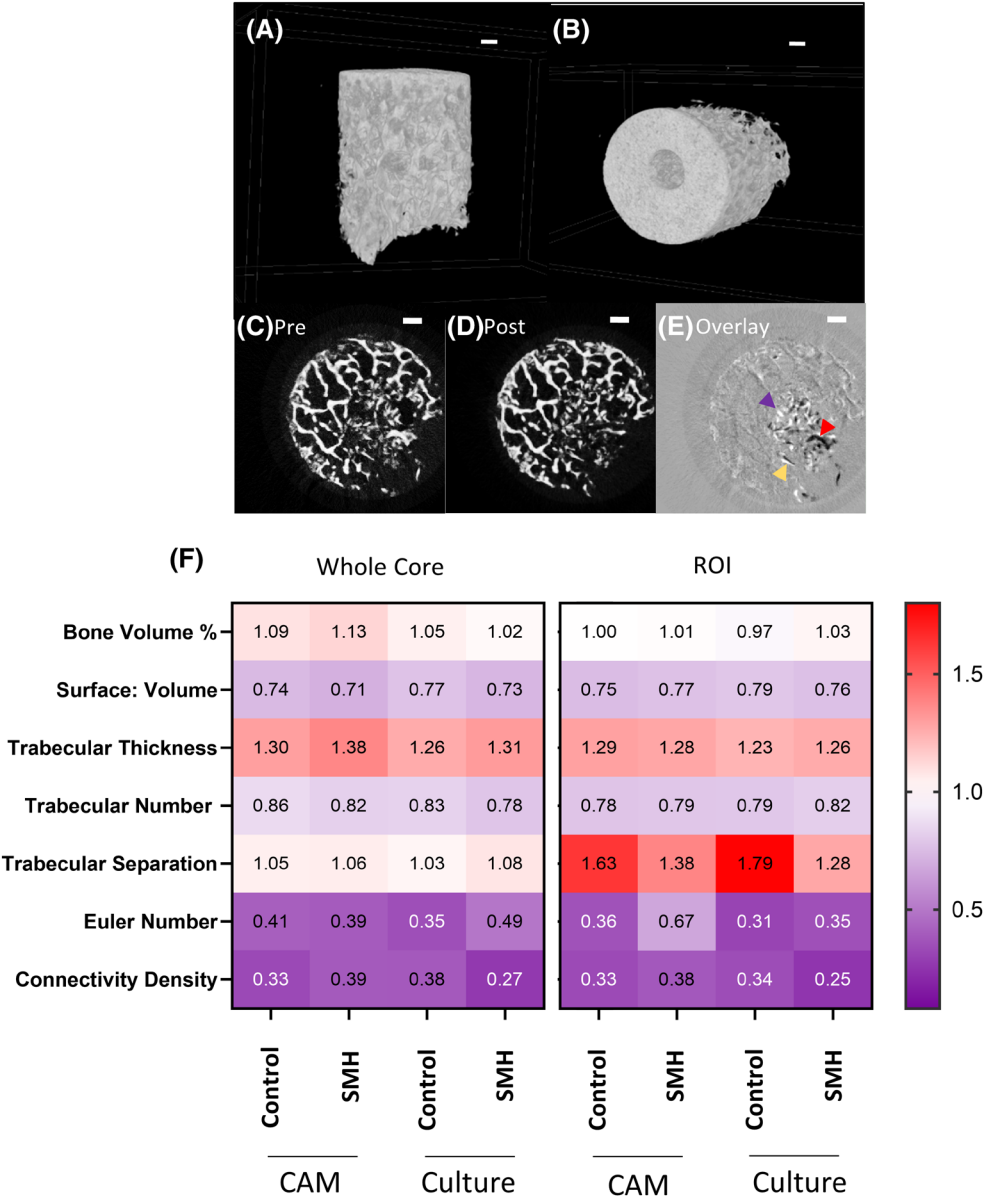
Micro‐CT analysis of the 3D bone cores. (A, B) Representative μCT images of a bone core. (C, D) Representative images pre and post incubation on the CAM, which were then overlaid in (E), where bone formation (red arrow), resorption (purple arrow), and areas of misalignment (yellow arrow) were identified. Scale bar = 1 mm. A combination of Saos‐2, MDMs and HBMSCs (abbreviated to SMH) were inserted into the bone cores before incubation in standard culture conditions or on the CAM for 11 days, with μCT images taken before and after culture. Analysis was performed on the whole bone core and a ROI approximately 1 mm around the defect area. (F) Heat maps represent the fold change, of percentage bone volume, bone surface to volume ratio, trabecular thickness, trabecular number, trabecular separation, Euler number and connectivity density. *N* = 3–5 biological replicates. Results presented as mean, statistics analyzed using a one‐way ANOVA, no significance identified.

Utilizing μCT imaging techniques enables visualization and quantification of early changes in bone remodeling. Figure 5 depicts representative images of bone cores after μCT analysis (A and B). These images can be taken before (Figure 5C) and after (Figure 5D) incubation then overlaid (Figure 5E) to show areas of bone formation (indicated with a red arrow), resorption (purple arrow), or misalignment (when the trabecular bone has shifted during incubation, indicated with a yellow arrow).

Various trabecular bone measurements were generated and this data was used to accurately evaluate bone development and remodeling. Analysis of the whole bone core alongside a region of interest (ROI) depicted approximately 1 mm around the defect area (identified as the tumor niche) were assessed. This included a suggested minimum set of variables needed to identify bone remodeling through μCT analysis.^29^ These variables comprise percentage bone volume, trabecular number, trabecular thickness, trabecular separation, and bone surface‐to‐volume ratio. These data were combined alongside other quantitative measurements including Euler number and connectivity density. Euler number compares the number of cavities and objects to calculate the number of connections needed to split the bone structure in two. Connectivity density measures the amount of connected bone divided by the total volume of the sample.^29^ The combination of all these variables identified whether during culture there were changes in the structure of the bone (bone formation or bone resorption). In Figure 5F, μCT analysis of bone cores incubated in standard or CAM culture indicated there were no significant differences in bone remodeling when the three cell types were combined and inserted into the bone core (data also in Table S1). This highlighted the similar viability of bone cores when incubated on the CAM compared to those incubated in optimal standard culture conditions. Analysis of bone cores incubated with only one of the cell types (Table S2) showed a significant increase in trabecular number in the ROI when incubated with Saos‐2 cells compared to the control, but no significant changes were seen in the remaining variables or other cell types.

### 3.4 The effect of mifamurtide on the 3D osteosarcoma model

**FIGURE 6:**
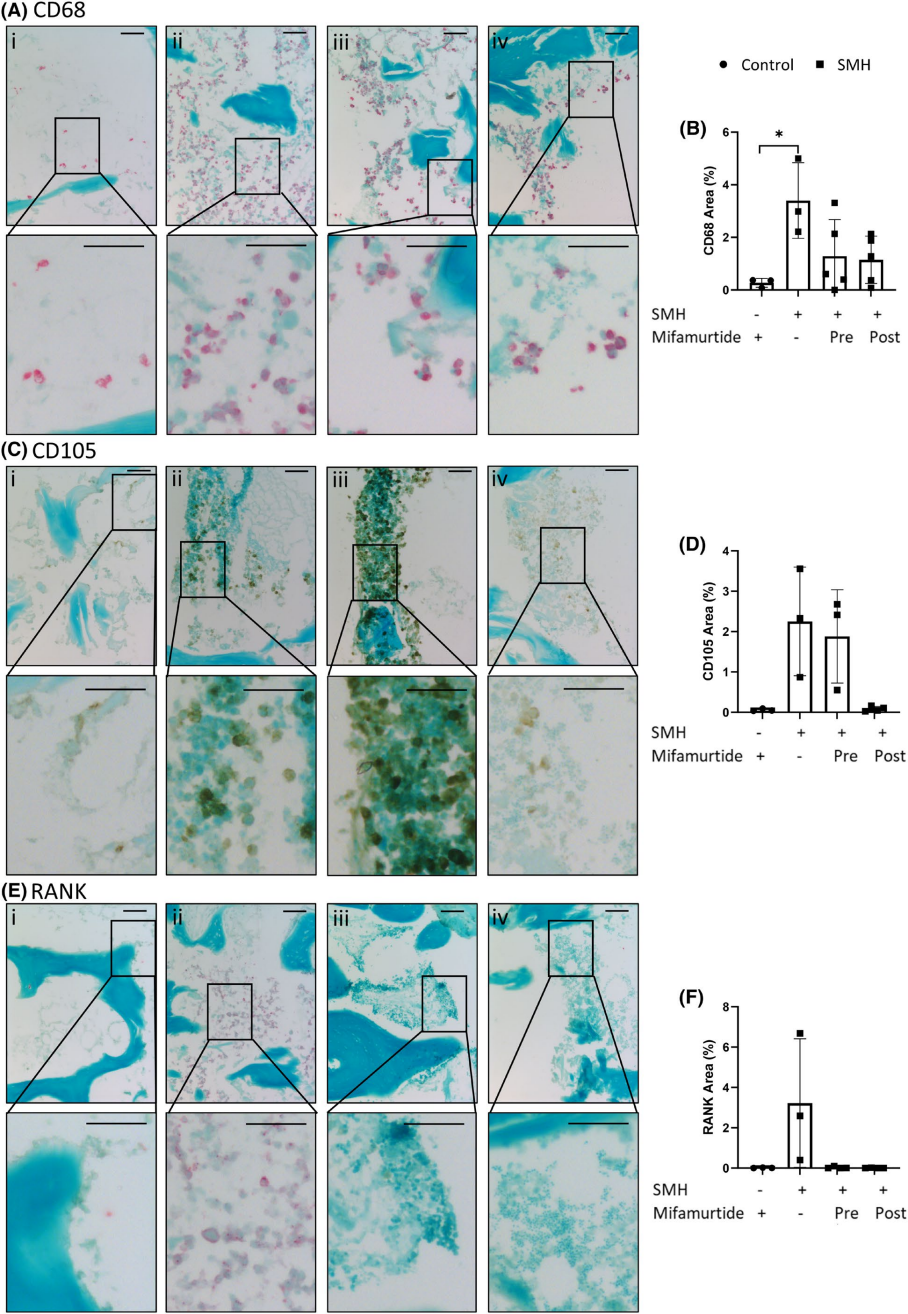
Characterization of the 3D bone cores after incubation with osteosarcoma drug mifamurtide. Bone cores were injected with Saos‐2 cells, MDMs and HBMSCs (SMH), implanted on the CAM for 11 days, and treated with mifamurtide. Representative images were taken, and the percentage area of expression was quantified for CD68 (A, B), CD105 (C, D) or RANK (E, F). (i) Control = cores with no inserted cells, (ii) Cells inserted with SMH, (iii) SMH bone cores treated with mifamurtide for 5 days before implantation, (iv) SMH bone cores treated with mifamurtide for five days after removal from the CAM. Scale bar = 50 μm. *N* = 3–5 biological replicates. Results presented as mean ± SD, statistics analyzed using a one‐way ANOVA, significance presented as *<.05.

We next sought to investigate if this 3D model had the potential to be used to test osteosarcoma drugs and therapies. Here, bone cores that contained a combination of the three cell types of interest (SMH) were either cultured for 5 days in mifamurtide before implantation in the CAM (Pre), to stimulate the cells before the vasculature was introduced. Or cultured with mifamurtide for 5 days after removal from the CAM (Post) when the model had been established (Figure S1). We determined not to directly inoculate the CAM with mifamurtide in this initial pilot study due to the unknown effect it may have on embryo growth and development. Empty bone cores were incubated in mifamurtide before implantation as an additional control. A final concentration of 0.16 μg/mL (6.4 μM) of mifamurtide was used to treat bone cores based on published data,^30^ and alamar blue analysis of HBMSCs (Figure S2). Representative images of CD68 expression in the four different conditions are shown in Figure 6A and this is quantified in Figure 6B. The percentage area of CD68 was significantly higher in the SMH bone cores compared to controls but this decreased when incubated with mifamurtide, both before and after implantation on the CAM. Interestingly, the level of CD105 (Figure 6C,D) was higher in SMH bone cores but only decreased when incubated with mifamurtide after removal from the CAM membrane. From this we inferred that there was either an overall decrease in endothelial cell number or in their proliferation, as CD105 is strongly expressed on proliferating endothelial cells.^28^ The percentage area of RANK (Figure 6E,F), a marker of osteoclasts, showed a similar pattern to the CD68 staining with an increase in the SMH bone cores and a reduction after incubation with mifamurtide.

**FIGURE 7:**
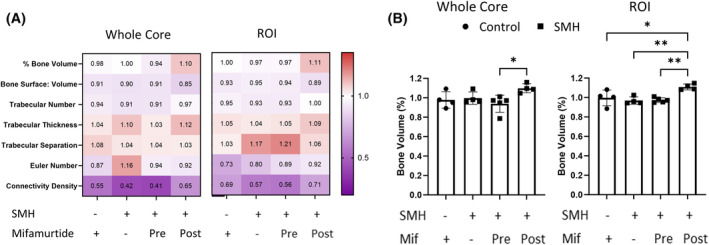
Micro‐CT analysis of 3D bone cores treated with mifamurtide. Bone cores were injected with a combination of Saos‐2 cells, MDMs and HBMSCs (SMH) and implanted on the CAM for 11 days. The bone cores were either incubated with mifamurtide for 5 days prior to implantation or for 5 days after removal from the CAM. μCT images were taken before and after incubation and analysis was performed on the whole bone core and a ROI approximately 1 mm around the defect area (A). (B) The percentage bone volume of the whole core and ROI. *N* = 3–5 biological replicates, Data presented as mean ± SD, for each parameter the statistics was determined using a one‐way ANOVA, significance represented as *<.05, **<.01.

Analysis of these bone cores using μCT (Figure 7A) also showed significant changes in bone volume for both the whole core and the ROI (Figure 7B), with an increase in bone volume when incubated with mifamurtide after removal from the CAM compared to the other conditions. This increase was more significant in the ROI compared to the whole bone core, suggesting phenotypic changes in the cells inserted into the defect area may be causing this shift in bone remodeling. Mean ± SD of the complete data set is contained in Table S3. While not significant, there were also trends seen in other parameters, for example, there was a higher trabecular number when the bone cores were incubated with mifamurtide after removal from the CAM, which support the premise that bone formation may have been occurring. The evidence highlighted in these experiments demonstrating active bone remodeling when treated with mifamurtide shows the benefit of developing a 3D bone model to test drugs and therapies for osteosarcoma. This could provide a better understanding of the mechanisms these drugs exert in the tumor microenvironment and the surrounding bone trabecular architecture.

## 4 DISCUSSION

Elucidating the complexities of the tumor microenvironment is critical for the continued development of successful new cancer treatments. Unfortunately, the osteosarcoma microenvironment is still poorly understood and therefore better, multifaceted models are urgently needed. While in vivo mouse studies allow for both systems level and more in‐depth investigations into the cellular interactions and inflammatory responses to tumor cells, the majority of bone cancer models are limited by the difficulty in either injecting into the bone or the sporadic development of bone tumors. Although the development of in vitro 3D models has been used to investigate various aspects of osteosarcoma growth and function (Table 1), few have been able to replicate the complexity of the tumor microenvironment. Scaffold free 3D models^31^, ^32^, ^33^, ^34^, ^35^, ^36^, ^37^ have increased our understanding of cancer cell proliferation and migration but have not been able to replicate the structural aspects of osteosarcoma biology including osteoid deposition. The development of hydrogel scaffolds in osteosarcoma research have become more prevalent, investigating invasiveness of tumor cells alongside drug development.^38^, ^39^, ^40^, ^41^, ^42^ Unfortunately, even with a large range of materials these hydrogels have not yet been able to fully recapitulate the functional bioactivity and mechanical stiffness of human bone.^7^, ^43^ The *in ovo* CAM model has been used in cancer research for many years, including for osteosarcoma,^12^, ^16^, ^17^, ^39^ and has been integral to investigation of tumor development, invasiveness and angiogenesis. The main limitation of published CAM models of osteosarcoma is their focus on the osteosarcoma cells themselves and not on combining other prominent cells and bone tissue integral to the tumor microenvironment.

**TABLE 1:** Published 3D models of osteosarcoma.

	Structure	Composition		Limitations
In vitro	Scaffold free	Hanging drop	31–33	Limited evidence of osteoid deposition
		Liquid overlay	34–37	
	Scaffold—Hydrogel	Collagen based	38, 39	Various compositions give differing results in mechanical stiffness, bioactivity, etc
		Gelatin based	40–42	
*In ovo*	CAM—Scaffold	Plastic rings	12	CAM models previously focused on osteosarcoma cells
		Sponges	16	
		Collagen/Matrigel	17, 39	

*Note*: In vitro and *in ovo* 3D models of osteosarcoma that have been published in peer‐reviewed journals.

To attempt to bridge this gap between scaffold development, in vivo models and the complexity of the tumor microenvironment we have developed a novel 3D bone model of osteosarcoma. This 3D bone model consisted of human bone cylinders impregnated with a mixture of Saos‐2, MDMs and HBMSCs, and incubated on the CAM of a fertilized chicken egg (Figure 1). The use of human bone more accurately replicated the 3D structure of the osteosarcoma microenvironment compared to alternative scaffolds (Table 1). Implanting the bone cores on the CAM resulted in angiogenesis (Figure 2), in part mimicking the vasculature system found in human tumors. The implantation of multiple cell types also allowed for investigation into the wider tumor microenvironment and cellular communication not previously found in CAM models of osteosarcoma. Furthermore, the inserted cells were identified through imaging techniques to remain within the defect area, allowing for the development of a tumor ‘niche’ of interacting cells (Figure 3).

Characterization of the bone cores by IHC staining of CD68 and CD105 (Figure 4A–H) showed a similar level of expression between bone cores incubated in standard culture conditions and the bone cores implanted on the CAM. This suggested that implanting the bone cores on the CAM did not negatively affect the viability of the cells in the bone model and that the successful integration of the chick vasculature system allowed for movement of nutrients and waste around the model to keep the cells viable. As the aim of the 3D bone model was to replicate aspects of the human osteosarcoma microenvironment, ten osteosarcoma patient samples were sectioned and stained for CD68 and CD105 (Figure 4I–L). These patient sections exhibited a range of expression for both markers, displaying patient variability. Notably, the level of CD68 and CD105 expressed in both the CAM and standard cultured 3D bone models were within the range identified in the patient sections. While CD105 was initially selected as a general marker for identifying HBMSCs in the bone model, to confirm insertion was successful, it became clear a more extensive selection of markers would better distinguish specific cell types including mesenchymal stem cells, osteoblast/osteoid markers to identify areas of bone development, and Ki67 to identify areas of cellular proliferation. It is known that osteosarcoma cells can differentiate down the trilineage pathway and this model has the potential to assess this in more detail, including changes in chondrogenic and adipogenic expression.

Irregular bone formation is often found in osteosarcoma development, and can be helpful in distinguishing osteosarcoma from other bone cancers.^44^ Identification of bone formation in the model was identified by μCT analysis, although large changes in bone remodeling could be limited by the relatively short incubation time on the CAM. The human bone used here to develop the 3D model allowed for resident stromal cells to be included in the bone structure, but as these bone tissue samples were acquired from patients with diseases like osteoporosis or osteoarthritis that could have affected the composition of these cells. For example, they may have an increased number of osteoclasts compared to healthy samples. As previously identified,^15^ HBMSCs isolated from the femoral epiphysis showed a different phenotype compared to those from the femoral diaphysis/metaphysis. Osteosarcoma occurs around growth plates,^1^ which are also sites of bone marrow conversion.^45^ By introducing HBMSCs isolated from the femoral diaphysis/metaphysis into the bone model that already contained bone marrow from the femoral epiphysis, we were effectively simulating this bone marrow conversion and change in phenotype, potentially reducing any impact from osteoporotic/ osteoarthritic disease.

As patient survival rates for osteosarcoma have shown little improvement over the last few decades,^1^, ^46^ the necessity to develop new treatments is a clear clinical objective. The 3D bone core model of osteosarcoma developed here has the potential to be a powerful tool in this area. These bone models have been treated with the drug mifamurtide, which resulted in reductions in the expression of CD68 (Figure 6A,D) and RANK (Figure 6B,E), as well as a reduction in CD105 when incubated with drug after removal from the CAM (Figure 6C,F). Although its method of action is still being investigated, it is understood that mifamurtide may work, in part, by binding to the nucleotide‐binding oligomerization domain 2 protein which induces nuclear factor kappa B to activate the immune response of phagocytic cells.^3^ Consequently, the reduction in the macrophage marker CD68 observed in our model could be due to the activation and subsequent death of these cells. Whether mifamurtide directly activates osteoclasts is unknown, but there has been evidence that mifamurtide might act as an anti‐resorption agent when combined with chemotherapy.^47^ As osteoclasts have a phagocytic ability, albeit centered on bone resorption, mifamurtide could directly or indirectly affect osteoclast function in the 3D bone model, resulting in a reduction of RANK expression. TRAP staining is an established method for identifying osteoclasts, unfortunately due to the high acidity required for decalcification this was not possible in our model and thus RANK was used as an alternative.

The marker CD105 is predominantly expressed on cells within the vasculature system particularly on proliferating endothelial cells,^28^ a cell type present in HBMSCs. Reduction of CD105 expression after mifamurtide treatment (post), could suggest that there was an overall decrease in the proliferation of endothelial cells compared to the mifamurtide treated (pre) bone cores. Treating the bone cores with mifamurtide after angiogenesis and removal from the CAM is arguably more clinically relevant. Thus, the reduction in CD105 and possible decrease in endothelial cell proliferation could be another effective target of mifamurtide. Furthermore, the 3D bone cores incubated in mifamurtide (post) showed a significant increase in bone volume (Figure 7), from both the whole core (B) and a ROI (C). While there were no significant differences in the other μCT variables assessed there was an increased trend in trabecular number and thickness compared to the control bone cores. With bone remodeling occurring after treatment with mifamurtide, it suggests that as well as being used to screen new treatments for osteosarcoma, this 3D bone model could also potentially be used to help investigate the method of action of mifamurtide.

While development of new drugs and therapies to treat osteosarcoma is needed, adequate and representative models to test these drugs are lacking. The 3D multicellular bone model of osteosarcoma established here has the potential to help meet this requirement. Utilizing the CAM induces vascularization of the bone model, which is an essential characteristic of human primary tumors and a factor currently not represented in standard culture conditions. By using human bone cores as the structural element of the model and implanting these on the CAM, viable tissue was recovered and showed similar characteristics to osteosarcoma patient sections. This 3D model has also shown the capacity to help us understand how the bone microenvironment can be altered and has the potential to serve as a platform to assess future osteosarcoma treatments.

## AUTHOR CONTRIBUTIONS

Conceptualization: Hannah L. Smith, Stephen A. Beers, Janos M. Kanczler, and Juliet C. Gray. Methodology: Hannah L. Smith, Stephen A. Beers, Janos M. Kanczler, and Juliet C. Gray. Investigation: Hannah L. Smith. Visualization: Hannah L. Smith. Funding acquisition: Stephen A. Beers, Janos M. Kanczler, and Juliet C. Gray. Project administration: Stephen A. Beers, Janos M. Kanczler, and Juliet C. Gray. Supervision: Stephen A. Beers, Janos M. Kanczler, and Juliet C. Gray. Writing—original draft: Hannah L. Smith. Writing—review & editing: Stephen A. Beers, Janos M. Kanczler, and Juliet C. Gray.

## DISCLOSURES

H. Smith and J. Kanczler have no conflicts of interest. S. Beers has consulted for Astex Pharmaceuticals, BioInvent, Epsilogen, F‐star Therapeutics, ImCheck Therapeutics and LTZ Therapeutics, and has received research funding from BioInvent and ImCheck Therapeutics. J. Gray has had consulting roles for EUSA Pharma, YmAbs Therapeutics, Celgene, Norgine and Servier, and has received research funding from Celgene/Bristol Myers Squibb and Eusa Pharma.

## Supporting information


Figure S1.

Figure S2.

Table S1.

Table S2.

Table S3.

## Data Availability

The data that support the findings of this study are available in the materials and methods, results and supplemental material of this article.
