# Supplementary material for: Developing a 3D bone model of osteosarcoma to investigate cancer mechanisms and evaluate treatments
Source: FASEB J. 2024 Dec 26;38(24):e70274. doi: 10.1096/fj.202402011R (PMC11670810; doi:10.1096/fj.202402011R)
Supplement: Supplementary file 1 — Figure S1. [file FSB2-38-e70274-s003.pdf]

SUPPLEMENTARY

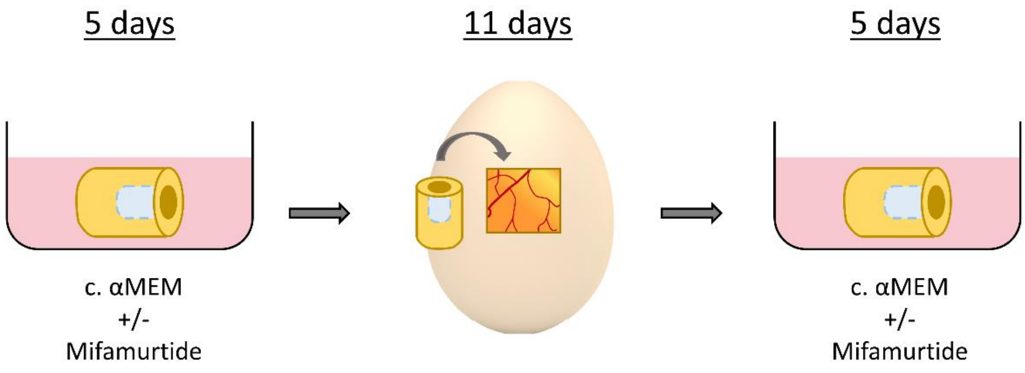

Supplementary Figure 1. Mifamurtide treatment of bone cores. Bone cores were cultured with c.αMEM media +/- 6.4 µM mifamurtide for 5 days, they were then incubated on the CAM for 11 days before being cultured with c.αMEM media +/- 6.4 µM mifamurtide for a final 5 days.
