# Supplementary material for: Developing a 3D bone model of osteosarcoma to investigate cancer mechanisms and evaluate treatments
Source: FASEB J. 2024 Dec 26;38(24):e70274. doi: 10.1096/fj.202402011R (PMC11670810; doi:10.1096/fj.202402011R)
Supplement: Supplementary file 2 — Figure S2. [file FSB2-38-e70274-s004.pdf]

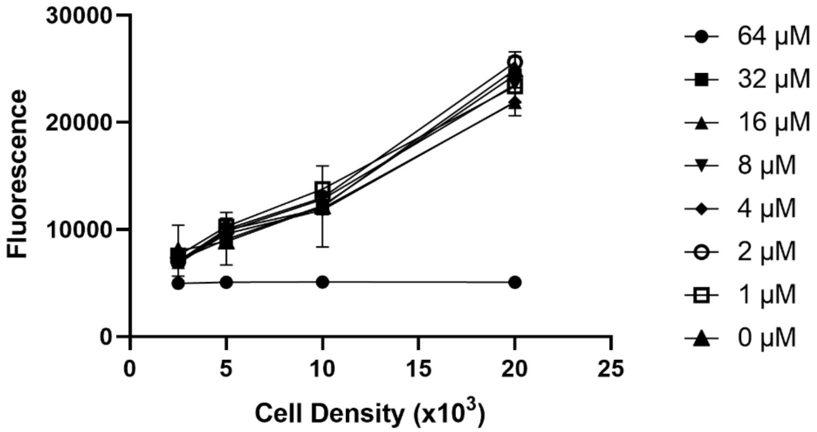

Supplementary Figure 2. Proliferation of HBMSCs incubated with mifamurtide for 24 hours. HBMSCs were plated into a 96 well plate and incubated with reducing concentrations of mifamurtide ranging from 0 to 64 μM. After 24 hours the cells were washed and incubated with alamar blue (diluted 1 in 10) for 1 hour before the fluorescence was measured at 560nm. No reduction was seen in the alamar blue fluorescence of the HBMSC proliferation except for the highest concentration of 64 μM. The concentration of 6.4 μM of mifamurtide (1:10 dilution) was chosen for bone core analysis in line with literature findings.
