# Supplementary material for: Developing a 3D bone model of osteosarcoma to investigate cancer mechanisms and evaluate treatments
Source: FASEB J. 2024 Dec 26;38(24):e70274. doi: 10.1096/fj.202402011R (PMC11670810; doi:10.1096/fj.202402011R)
Supplement: Supplementary file 3 — Table S1. [file FSB2-38-e70274-s001.pdf]

		CAM		Culture	
		Control	SMH	Control	SMH
**Bone Volume %**	Whole Core	1.09 ± 0.06	1.13 ± 0.13	1.05 ± 0.04	1.02 ± 0.04
	ROI	1.00 ± 0.05	1.01 ± 0.06	0.97 ± 0.08	1.03 ± 0.06
**Bone Surface: Volume**	Whole Core	0.74 ± 0.03	0.71 ± 0.06	0.77 ± 0.03	0.73 ± 0.04
	ROI	0.75 ± 0.05	0.77 ± 0.03	0.79 ± 0.06	0.76 ± 0.04
**Trabecular Thickness**	Whole Core	1.23 ± 0.05	1.38 ± 0.07	1.26 ± 0.03	1.31 ± 0.05
	ROI	1.29 ± 0.07	1.28 ± 0.03	1.23 ± 0.06	1.26 ± 0.03
**Trabecular Number**	Whole Core	0.86 ± 0.04	0.82 ± 0.09	0.83 ± 0.05	0.78 ± 0.06
	ROI	0.78 ± 0.08	0.79 ± 0.05	0.79 ± 0.11	0.82 ± 0.05
**Trabecular Separation**	Whole Core	1.05 ± 0.02	1.06 ± 0.02	1.03 ± 0.02	1.08 ± 0.04
	ROI	1.63 ± 0.64	1.38 ± 0.15	1.79 ± 0.48	1.28 ± 0.24
**Euler Number**	Whole Core	0.41 ± 0.05	0.39 ± 0.11	0.35 ± 0.03	0.49 ± 0.02
	ROI	0.36 ± 0.09	0.67 ± 0.28	0.31 ± 0.10	0.35 ± 0.19
**Connectivity Density**	Whole Core	0.33 ± 0.06	0.39 ± 0.12	0.38 ± 0.02	0.27 ± 0.10
	ROI	0.33 ± 0.08	0.38 ± 0.09	0.34 ± 0.15	0.25 ± 0.01

Supplementary Table 1. Micro-CT analysis of bone cores inoculated with Saos-2, MDMs and HBMSCs (SMH) and cultured on the CAM or in standard culture conditions for 11 days. N=2-5 biological replicates, Data presented as mean +/- SD.
