# Supplementary material for: Developing a 3D bone model of osteosarcoma to investigate cancer mechanisms and evaluate treatments
Source: FASEB J. 2024 Dec 26;38(24):e70274. doi: 10.1096/fj.202402011R (PMC11670810; doi:10.1096/fj.202402011R)
Supplement: Supplementary file 4 — Table S2. [file FSB2-38-e70274-s005.pdf]

		Control	Saos-2	MDM	HBMSC
**Bone Volume %**	Whole Core	1.09 ± 0.06	1.06 ± 0.07	1.09 ± 0.14	1.08 ± 0.03
	ROI	1.00 ± 0.05	1.08 ± 0.04	1.05 ± 0.04	1.06 ± 0.02
**Bone Surface: Volume**	Whole Core	0.74 ± 0.03	0.81 ± 0.05	0.71 ± 0.04	0.74 ± 0.07
	ROI	0.75 ± 0.05	0.84 ± 0.04	0.76 ± 0.06	0.78 ± 0.09
**Trabecular Thickness**	Whole Core	1.30 ± 0.05	1.21 ± 0.08	1.36 ± 0.06	1.30 ± 0.10
	ROI	1.29 ± 0.07	1.18 ± 0.06	1.29 ± 0.07	1.27 ± 0.12
**Trabecular Number**	Whole Core	0.86 ± 0.04	0.88 ± 0.03	0.80 ± 0.03	0.83 ± 0.06
	ROI	0.78 ± 0.08	0.92 ± 0.04	0.82 ± 0.04	0.84 ± 0.07
**Trabecular Separation**	Whole Core	1.05 ± 0.03	1.06 ± 0.03	1.06 ± 0.01	1.05 ± 0.04
	ROI	1.17 ± 0.03	1.02 ± 0.07	1.19 ± 0.16	1.01 ± 0.38
**Euler Number**	Whole Core	0.41 ± 0.05	0.42 ± 0.26	0.41 ± 0.07	0.39 ± 0.08
	ROI	0.36 ± 0.09	0.22 ± 0.01	0.23 ± 0.11	0.34 ± 0.10
**Connectivity Density**	Whole Core	0.33 ± 0.06	0.36 ± 0.14	0.35 ± 0.14	0.38 ± 0.17
	ROI	0.33 ± 0.08	0.78 ± 0.05	0.34 ± 0.07	0.50 ± 0.23

Supplementary Table 2. Micro-CT analysis of bone cores inoculated with individual cell types. N=3-5 biological replicates, Data presented as mean +/- SD.
