# Supplementary material for: Developing a 3D bone model of osteosarcoma to investigate cancer mechanisms and evaluate treatments
Source: FASEB J. 2024 Dec 26;38(24):e70274. doi: 10.1096/fj.202402011R (PMC11670810; doi:10.1096/fj.202402011R)
Supplement: Supplementary file 5 — Table S3. [file FSB2-38-e70274-s002.pdf]

		Control	SMH	SMH +Pre	SMH +Post
**Bone Volume %**	Whole Core	0.98 ± 0.07	1.00 ± 0.06	0.94 ± 0.08	1.10 ± 0.04
	ROI	1.00 ± 0.07	0.97 ± 0.03	0.97 ± 0.02	1.11 ± 0.03
**Bone Surface: Volume**	Whole Core	0.92 ± 0.05	0.90 ± 0.04	0.91 ± 0.04	0.85 ± 0.03
	ROI	0.93 ± 0.05	0.95 ± 0.02	0.94 ± 0.01	0.89 ± 0.01
**Trabecular Thickness**	Whole Core	1.04 ± 0.04	1.10 ± 0.09	1.03 ± 0.02	1.12 ± 0.03
	ROI	1.05 ± 0.04	1.04 ± 0.02	1.05 ± 0.02	1.09 ± 0.02
**Trabecular Number**	Whole Core	0.94 ± 0.03	0.91 ± 0.08	0.91 ± 0.08	0.97 ± 0.03
	ROI	0.95 ± 0.07	0.93 ± 0.02	0.93 ± 0.02	1.00 ± 0.04
**Trabecular Separation**	Whole Core	1.08 ± 0.06	1.04 ± 0.03	1.04 ± 0.02	1.03 ± 0.02
	ROI	1.03 ± 0.13	1.17 ± 0.10	1.21 ± 0.08	1.06 ± 0.13
**Euler Number**	Whole Core	0.87 ± 0.18	1.16 ± 0.29	0.94 ± 0.20	0.92 ± 0.30
	ROI	0.73 ± 0.21	0.80 ± 0.05	0.89 ± 0.21	0.92 ± 0.10
**Connectivity Density**	Whole Core	0.55 ± 0.14	0.42 ± 0.09	0.41 ± 0.15	0.65 ± 0.04
	ROI	0.69 ± 0.19	0.57 ± 0.05	0.56 ± 0.07	0.71 ± 0.11

Supplementary Table 3. Micro-CT analysis of bone cores inoculated with Saos-2, MDMs and HBMSCs (SMH) and incubated with or without mifamurtide for five days before (pre) implantation on the CAM or after (post) removal from the CAM. N=4-5 biological replicates, Data presented as mean +/- SD.
